# The Metabolic Basis of ILC Plasticity

**DOI:** 10.3389/fimmu.2022.858051

**Published:** 2022-04-29

**Authors:** Abigaelle Pelletier, Christian Stockmann

**Affiliations:** Institute of Anatomy, Faculty of Medicine, University of Zurich, Zurich, Switzerland

**Keywords:** Innate Lymphoid Cells (ILCs), metabolism, plasticity, OxPhos, glycolysis, cytokines

## Abstract

Innate Lymphoid Cells (ILCs) are the innate counterpart of adaptive lymphoid T cells. They are key players in the regulation of tissues homeostasis and early inflammatory host responses. ILCs are divided into three groups, and further subdivided into five subsets, that are characterised by distinct transcription factors, surface markers and their cytokine expression profiles. Group 1 ILCs, including natural killer (NK) cells and non-NK cell ILC1s, express T-bet and produce IFN-γ. Group 2 ILCs depend on GATA3 and produce IL-4, IL-5 and IL-13. Group 3 ILCs, composed of ILC3s and Lymphoid Tissue Inducer (LTi) cells, express RORγt and produce IL-17 and IL-22. Even though, the phenotype of each subset is well defined, environmental signals can trigger the interconversion of phenotypes and the plasticity of ILCs, in both mice and humans. Several extrinsic and intrinsic drivers of ILC plasticity have been described. However, the changes in cellular metabolism that underlie ILC plasticity remain largely unexplored. Given that metabolic changes critically affect fate and effector function of several immune cell types, we, here, review recent findings on ILC metabolism and discuss the implications for ILC plasticity.

## Introduction

Innate Lymphoid Cells (ILCs) are a relatively new population of immune cells which was discovered less than fifteen years ago. Several papers demonstrated the existence of natural killer (NK) cell-like immune cells that could be defined by their secretion profiles, similarly to lymphocyte T cells (1–5). Over the years, ILC populations were more precisely defined and five different subsets were described on the basis of lineage-defining transcription factor expression and cytokine secretion profiles (**Table 1**): group 1 ILCs (ILC1s), composed of NK cells and non-cytotoxic ILC1s, group 2 ILCs (ILC2s) and group 3 ILCs (ILC3s), composed of lymphoid tissue inducers (LTi) and “conventional” ILC3s (6–9). ILC1s are characterized by their expression of the transcription factor T-bet, and eomesodermin (EOMES)in the NK cell subset, and produce interferon-γ (IFN-γ) (6, 9, 10). ILC2s express GATA-binding protein 3 (GATA3) and can produce interleukin (IL)-4, IL-5 and IL-13 (5, 6, 9). Finally, ILC3s depend on retinoic acid receptor-related orphan receptor-γt (RORγt) and can produce IL-17 and IL-22 (6, 9, 11, 12). Of note, group 1 ILCs and a subset of ILC3s share the expression of the surface marker NKp46.

**Table 1:** Main phenotypic markers defining the different murine ILC subsets.

ILC group	ILC subset	Surface Marker	Transcription Factor	Secreted Cytokine
1	NK cell	NKp46	T-bet	IFN-γ
		NK1.1	EOMES	TNFα
		CD49b		Cytotoxic granules
	ILC1	NKp46	T-bet	IFN-γ
		NK1.1	HOBIT	TNFα
		CD49a		
2	ILC2	ICOS	GATA3	IL-4
		IL-17Rβ	RORα	IL-5
				IL-9
				IL-13
				AREG
3	ILC3	NKp46+	RORγt	IL-22
			Tbet	GM-CSF
			AhR	
		NKp46-	RORγt	IL-17
			AhR	IL-22
				IFN-γ
				GM-CSF
	LTi	c-Kit	RORγt	IL-17
		CCR6		IL-22
				Lymphotoxin

Similar to T cells, increasing evidences show that ILC phenotypes are not as rigid as first thought and that one subset can adapt the functions and phenotype of another subset (13). This phenomenon is called plasticity, which was first introduced by Helen Blau in 1985 to define the capacity of a cell to change its identity (14). Plasticity of mature ILCs has been demonstrated during tissue inflammation and cancer progression in mice and humans (15–23), and reproduced *in vitro* by several teams. Briefly, it has been shown that ILC3s are able to transdifferentiate into ILC1s when stimulated with IL-1β and IL-12 (24–30). Similarly, the interconversion of ILC2s into ILC1s was shown to be driven by the combination of IL-1β and IL-12 but also by IL-18 and IL-12 (15, 22, 23, 31). NK cells are able to convert to non-cytotoxic ILC1-like cells under the stimulation of transforming growth factor (TGF)-β and IL-12 (18, 20, 32, 33). In turn, ILC1s and ILC2s can adapt the phenotype of ILC3s when stimulated with IL-1β and IL-23 (17, 21, 28, 31). In the gut, where ILC3s are the most abundant followed by ILC2s, IL-12 upregulates T-bet expression while IL-1β induces the production of IFN-γ (16, 22) which drives the conversion of ILC3s and ILC2s into ILC1s. Similarly, in the lung where ILC2s are dominant, IL-12 and IL-1β drive the plasticity of ILC2s towards ILC1s (15, 23). A conversion of ILC2s into ILC3s was also shown in the lung where IL-23 and TGFβ control the upregulation of RORγt, the downregulation of GATA3 and the production of IL-17 in ILC2s (21, 34). Thus, plasticity of ILC subsets depends on complex cytokine combinations, with some cytokines being involved in the conversion of more than one subset. However, apart from transcription factors and cytokines such as TGF-β, very little is known about additional drivers of ILC plasticity, e.g. other microenvironmental stimuli or changes in ILC metabolism.

It is widely accepted that ILCs are the innate counterpart of T cells, ILC1 mirroring CD4^+^ T helper (Th) 1, ILC2 mirroring Th2 and ILC3 mirroring Th17 subset because of their similar function and secretion profiles (8). Plasticity of T helper subset are more extensively studied and it was shown that not only cytokines can trigger plasticity but also other key processes such as cellular metabolism (35–37). Thus, metabolism is likely to play a role in the plasticity of ILCs as well. In this review we describe what is known about the metabolism of human and mouse ILCs and we discuss the implication of metabolic changes for ILC plasticity.

## Metabolism of Innate Lymphoid Cells

Metabolism is critical for the survival, proliferation and function of all living cells. Several metabolic pathways fulfil the energy and biosynthetic demands of a cell. Energy, produced in the form of adenosine triphosphate (ATP), is generated by glycolysis and oxidative phosphorylation (OXPHOS). During glycolysis, glucose is converted to pyruvate by a series of reactions to produce ATP. Pyruvate can be converted to lactate or enter the mitochondria for OXPHOS. In addition to pyruvate, OXPHOS can also be fuelled by other carbon sources such as fatty acids and glutamine. OXPHOS generates reduced cofactors such as NADH, which are then oxidized in the electron transport chain to produce energy. These pathways, and the metabolic capacity of a cell, are tightly regulated by cell-intrinsic as well as -extrinsic factors. The last years have seen the emergence of a scientific discipline which studied the role of metabolism on the development, proliferation and function of immune cells, called immunometabolism. Whereas it has been widely studied in lymphocyte T cells, the adaptive counterpart of ILCs, a few is known about the metabolism of ILCs.

### NK cells

NK cells in a steady or quiescent state have a low metabolic need and rely mainly on the most efficient glucose-metabolizing pathway, the oxidative phosphorylation, to meet their metabolic demand. Upon activation, NK cells start to produce IFN-γ, perforin and granzyme B in order to kill their targets, changing their metabolic needs (38, 39).

During long-term stimulation (> 18 hours), NK cell metabolism increases and switches from OXPHOS metabolism to an aerobic glycolysis where pyruvate is degraded into lactate rather than entering the mitochondria (40–44). This metabolic reprogramming plays a crucial role in the cytotoxicity of NK cells as inhibiting this metabolic switch impairs NK effector function. It has been shown that the inhibition of glycolysis reduces IFN-γ production during activation as well as the number of granzyme B-expressing NK cells. This effect is partially regulated by the mammalian target of rapamycin complex 1 (mTORC1) transcription factor which was shown to be critical for human and mouse NK cell function (45–47). Indeed, mTORC1inhibition results in a reduced glycolysis, nutrient uptake and IFN-γ production (41). Expression of the transcription factor Srebp is increased when NK cells are stimulated with IL-2 and IL-12, which augments the amount of key components of the citrate-malate shuttle (CMS). The citrate-malate shuttle controls the exchange of mitochondrial citrate for a cytoplasmic malate, allowing the recycling of NAD to sustain a high OXPHOS and glycolytic rate (40). Inhibition of cMyc transcription factor also impairs glycolysis and OXPHOS in cytokine-stimulated NK cells. The study also shows the critical role of SLC7A5, an amino acid transporter, and glutamine. Inhibition of SLC7A5 activity or culture of NK cell in a glutamine-free medium lower the respiration, glycolysis and IFN-γ and granzyme B production (43). Surprisingly, this study showed that Hypoxia Inducible Factor (HIF)-1α plays no role in the metabolism of activated NK cells (43), even if its role on metabolism is well described in the literature. However, others studies investigating the role of HIF-1α in NK cell placed in hypoxic conditions show different results. Hypoxia, together with IL-15 or IL-12 and IL-18 stimulation, significantly upregulates glycolysis, OXPHOS and improves NK cell function by priming glycolytic genes transcription (48, 49). Moreover, Ni et al. showed that deletion of HIF-1α changes the metabolism of NK cells after seven days in hypoxia, decreasing aerobic glycolysis in quiescent NK cells and increasing OXPHOS in activated NK cells (48). At the same time, HIF-1α deletion drastically reduces NK cell killing capacities under hypoxia and normoxia when challenged with target cells, whereas NK cell activation upon cytokine stimulation and in the absence of target cells remains unaffected (48, 50).

During short-term stimulation (4-6 hours), however, the production of IFN-γ is not accompanied by any metabolic change. In this context, respiration and glycolysis stay low but they play a crucial role in mouse NK cell effector function. It has been shown that inhibition of either OXPHOS by oligomycin or glycolysis by 2-deoxy glucose (2-DG) impairs IFN-γ expression upon NK1.1-stimulation. On the other hand, IL-12 and IL-18-stimulated NK cells rely only on OXPHOS in short-term stimulation in order to produce IFN-γ (51).

**Figure 1:**
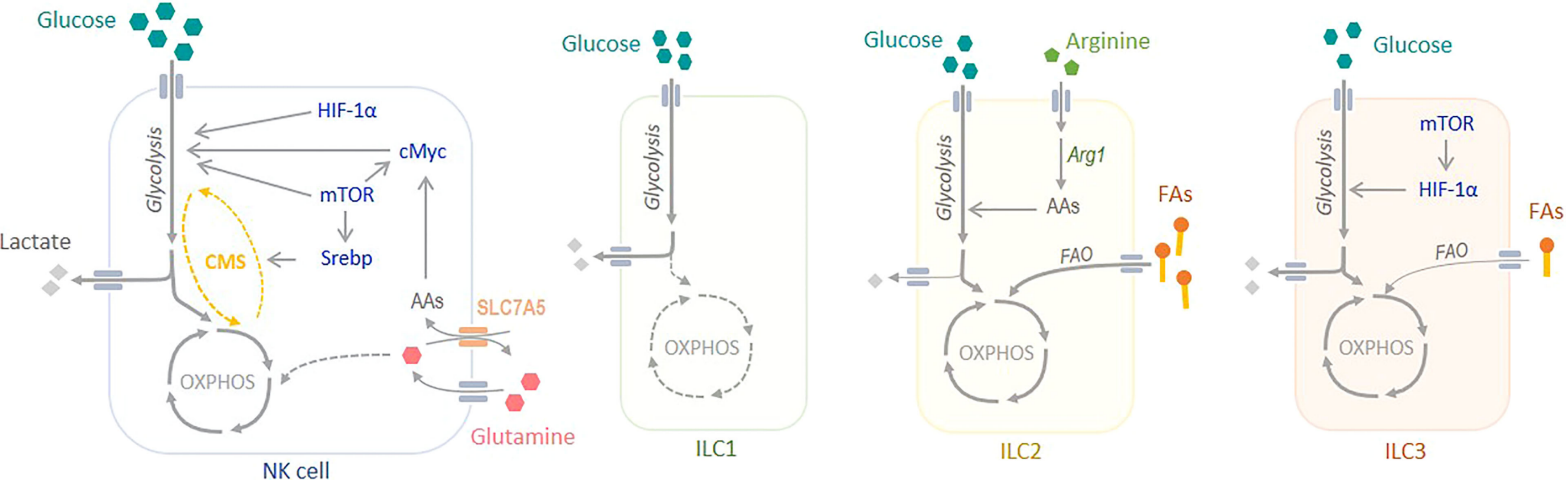
Metabolism of Innate Lymphoid Cells. NK cells rely mainly on glucose to meet their metabolic demand. At steady state, glucose sustains OXPHOS, whereas during activation it sustains aerobic glycolysis. Several transcription factors, such as mTORC1, Srebp, cMyc and HIF-1α play a critical role in regulating NK cell metabolism. The metabolism of non-cytotoxic ILC1s is poorly understood, but there are evidences showing an important role of glycolysis to sustain their metabolic demand. ILC2s rely mostly on OXPHOS and fatty acid oxidation (FAO) to produce energy. Arginase 1 (Arg1) is a key regulator of glycolysis and the amino acid (AA) pool in ILC2s. Finally, ILC3s obtain their energy from both OXPHOS, fuelled by glucose but also by fatty acids (FAs), and glycolysis. mTORC1 and HIF-1α are crucial in the regulation of ILC3 metabolism. CMS, citrate-malate shuttle.

In conclusion, likewise other immune cells, metabolism is crucial for NK cell survival, activation as well as effector function. Several transcription factors such as HIF-1α, mTORC1, cMyc and Srebp play a critical role in sustaining the metabolic needs for NK cell killing (**Figure 1**).

### Non-cytotoxic ILC1

The ILC1 subset is composed of the NK cells and the non-cytotoxic ILC1s, often called ILC1s. Whereas the metabolism of NK cells is a hot topic for their role in cancer, the metabolism of non-cytotoxic ILC1s is poorly studied. Often, the metabolism of this subgroup is assimilated to NK cell metabolism as they are both part of the ILC1 group. A comprehensive study on transcriptomic analysis of resting mouse ILCs shows that non-cytotoxic ILC1s are enriched in mTOR and Notch signaling pathways, suggesting an important role of glycolysis in their function (52), similarly to NK cells (**Figure 1**).

### ILC2

In a steady or quiescent state, mouse ILC2s rely mainly on Fatty Acid Oxidation (FAO) to fuel OXPHOS and meet their energetic demand. It has been shown that at steady state, mouse ILC2s from different tissues uptake a high amount of fatty acids *in vivo* compared to Treg, a T cell subset known to use FAO, ILC1s and ILC3s (53). Moreover, forcing a switch from fatty acid fuelled OXPHOS to aerobic glycolysis leads to a defect in ILC2 maturation and function, highlighting the importance of FAO in ILC2. Inhibition of FAO by etomoxir is sufficient to block ILC2 activation and cytokines, such as IL-5 and IL-13, secretion (54). Li et al. also showed that switching ILC2 metabolism, by deletion of von Hippel-Lindau (VHL) protein, has a strong impact on ILC2 development and effector function. VHL deletion induces a decrease in cellular respiration together with an increase aerobic glycolysis in a HIF-1α-dependent manner, leading to an impaired ILC2 development, maturation and cytokine secretion. Treatment with 2-DG, which inhibits glycolysis, rescues this phenotype (Q. 55). Consistently, blockade of FAO during infection and vitamin A deprivation impairs ILC2 accumulation and cytokine secretion (53).

Mouse activated ILC2s have a high metabolic potential and increase glycolysis, together with OXPHOS to meet their metabolic need upon activation (56). Indeed, Monticelli et al. compared activated murine ILC2s with their adaptative counterpart Th2 and showed that they have a higher spare respiratory capacity and glycolytic capacity, which correspond to the maximum oxidative phosphorylation rate and glycolysis rate of the cell, respectively (57). The same study highlights the key role of arginase 1 (Arg1) in the control of human and murine ILC2 metabolism and immune response, which was confirmed by Surace et al. later on (56). The absence of Arg1 diminishes the metabolic capacity of ILC2s by reducing their glycolytic capacity, without modifying OXPHOS, which reduces ILC2s immune response, proliferation and cytokine secretion (57). Interestingly, inhibition of Arg1 in lung ILC2 disrupts the amino acid (AA) balance by increasing the level of arginine and decreasing the levels of ornithine, proline and polyamines, which seem to play an important role in ILC2 metabolism (52, 57). A recent study on human circulating ILC2s showed that these cells are strongly dependent on OXPHOS when naïve. Surprisingly, their results suggest that ILC2s rely on branched chain amino acids (BCAAs) and arginine to fuel OXPHOS but not on FAO (56).

Thus, it is clear that ILC2s have a strong preference in OXPHOS to sustain their metabolic need during development, proliferation and activation (**Figure 1**). However, it seems that the sources of carbon used to fuel the Krebs cycle may vary between tissue resident and circulating ILC2s.

### ILC3

At steady state, ILC3s take up fatty acids to a lower extent than ILC2s but similar to ILC1s, and glucose to a similar extent than ILC2s (53). Upon activation, they increase their metabolism which is needed for an efficient secretion of cytokines (58). Analysis of the transcriptome of ILC3s confirms the key role of glycolysis in ILC3 as it highlights their enrichment in pathways associated with carbohydrates metabolism, such as glycolysis and pyruvate, galactose, fructose and mannose metabolisms (52). Indeed, mTORC1 and HIF-1α, two transcription factors controlling metabolism, play an important role in ILC3 development and functions. In animals with a specific deletion of mTORC1 subunit Raptor, Nkp46^+^ ILC3 number is reduced in the gut and they produce less IL-17 and IL-22 (47, 58).

Activation with IL-23 and IL-1β increases the metabolism of ILC3s compared to resting ILC3s in mice and humans (58, 59). It increases glucose and fatty acid uptake as well as glycolysis and OXPHOS. This change is controlled by the mTORC1-HIF-1α axis, as inhibiting one or the other suppresses this metabolic increase. As a consequence of increased OXPHOS, activated ILC3s also show an increased reactive oxygen species (ROS) level in the mitochondria, which is rescued by rapamycin, an mTOR inhibitor. Moreover, the increased glycolysis, OXPHOS and mitochondrial ROS induced by mTORC-HIF-1α play a crucial role in cytokine secretion and proliferation (58). This result was confirmed by Fachi et al. who showed that inhibiting glycolysis reduces the expression of RORγt and the secretion of IL-22 and IL-17 (59).

ILC3s are dominant in the gut, where they are directly influenced by numerous metabolites derived from food intake and by the hypoxic environment. Vitamin A is one of the key food-derived metabolites influencing ILC3 accumulation and function. Indeed, vitamin A deficiency in mice leads to a reduction in ILC3 number in the gut, IL-22 production and RORγt expression, together with an increased susceptibility to bacterial infection (60–62). Similarly, differentiation of LTi in the foetus is dependent on the maternal intake of vitamin A (63). Moreover, the hypoxic condition of the gut also promotes ILC3s proliferation and function at the expense of ILC1s (59, 64, 65).

Unlike the ILC1s and the ILC2s which rely either on glycolysis or on OXPHOS for their maturation and function, ILC3s seem to use both metabolic pathways to fulfil their need (**Figure 1**). It has been shown that OXPHOS and glucose oxidation are necessary. Moreover, their predominance in the gut make them influenced by vitamin A from the diet and hypoxia.

## Role of Metabolism on ILC Plasticity

### ILC3 and ILC1

It was shown that hypoxia and HIF-1α have a strong impact on ILC balance in the gut. HIFs are a family of dimeric transcription which respond to changes in oxygen level. The two subunits of HIF are constitutively expressed in cells, but HIF-1β is maintained at a constant level whereas HIF-1α concentration depends on oxygen (66, 67). Under normoxia, the α-subunit is hydroxylated by oxygen-sensitive enzymes, prolyl hydroxylase domain enzymes (PHDs) and factor-inhibiting HIF-1 (FIH-1). These enzymes hydroxylate one or the two prolyl residues present on the α-subunit, which leads to the creation of a binding site for VHL. Binding of VHL leads to HIFα ubiquitination and its proteasomal degradation. Under hypoxia, the hydroxylases are inhibited by the absence of oxygen, the α-subunit is not degraded and it accumulates. HIFα binds to the β-subunit and form an active complex that will translocate to the nucleus and then binds to its target genes which are involved in several important processes (68, 69). HIF-1α plays a key role in the control of catabolism in cells. Indeed, it increases the production of glucose transporters GLUT1 and GLUT3, and of hexokinase, the rate-limiting enzyme of the glycolysis (70). HIF-1α also regulates LDHA, which is responsible for the conversion of pyruvate into lactate, and monocarboxylate transporter 4, which evacuates lactate out of the cell (70, 71). Moreover, pyruvate dehydrogenase kinase 1 (PDK1), an enzyme which phosphorylates and inactivates PDH, is also regulated by HIF-1α. Consequently, flux of acetyl-CoA derivated from glucose is reduced (72). Thus, HIF-1 transcription factor is able to control the fate of glucose and redirects its use into anaerobic glycolysis instead of OXPHOS pathway by increasing LDHA activity and inhibiting PDH activity.

In mice, deletion of HIF-1α in RORγt expressing cells decreases ILC3 numbers while increasing ILC1s in the colon and in the small intestine. At the contrary, moderate hypoxia (8% O_2_) exposure of mice decreases the number of ILC1s and increases ILC3s in the small intestine lamina propria. Interestingly, the same study shows that hypoxia increases RORγt expression, and IL-17 and IL-22 secretion, while decreasing T-bet expression and IFN-γ secretion in ILC3s. Consistently, deletion HIF-1α in RORγt^+^ ILC3s lead to higher T-bet expression and decreased RORγt levels, along with a reduction of IL-22^+^ and IL-17^+^ ILC3s (59). However, RORγt-driven deletion of HIF-1α does not allow to discriminate between NKp46^−^ and NKp46^+^ ILC3s. Interestingly, a recent study from our lab demonstrated that loss of HIF-1α in NKp46^+^ cells leads to an increased NKp46^+^ ILC3/ILC1 ratio, accompanied by a decrease in IFN-γ in ILC1s and an increase in IL-22 in NKp46^+^ ILC3s. We also show that HIF-1α promotes ILC3-to-ILC1 conversion while inhibiting ILC1-to-ILC3 conversion *in vitro* and *in vivo* through direct transcriptional upregulation of *Tbx21* (the gene that encodes for T-bet) (73).

**Figure 2:**
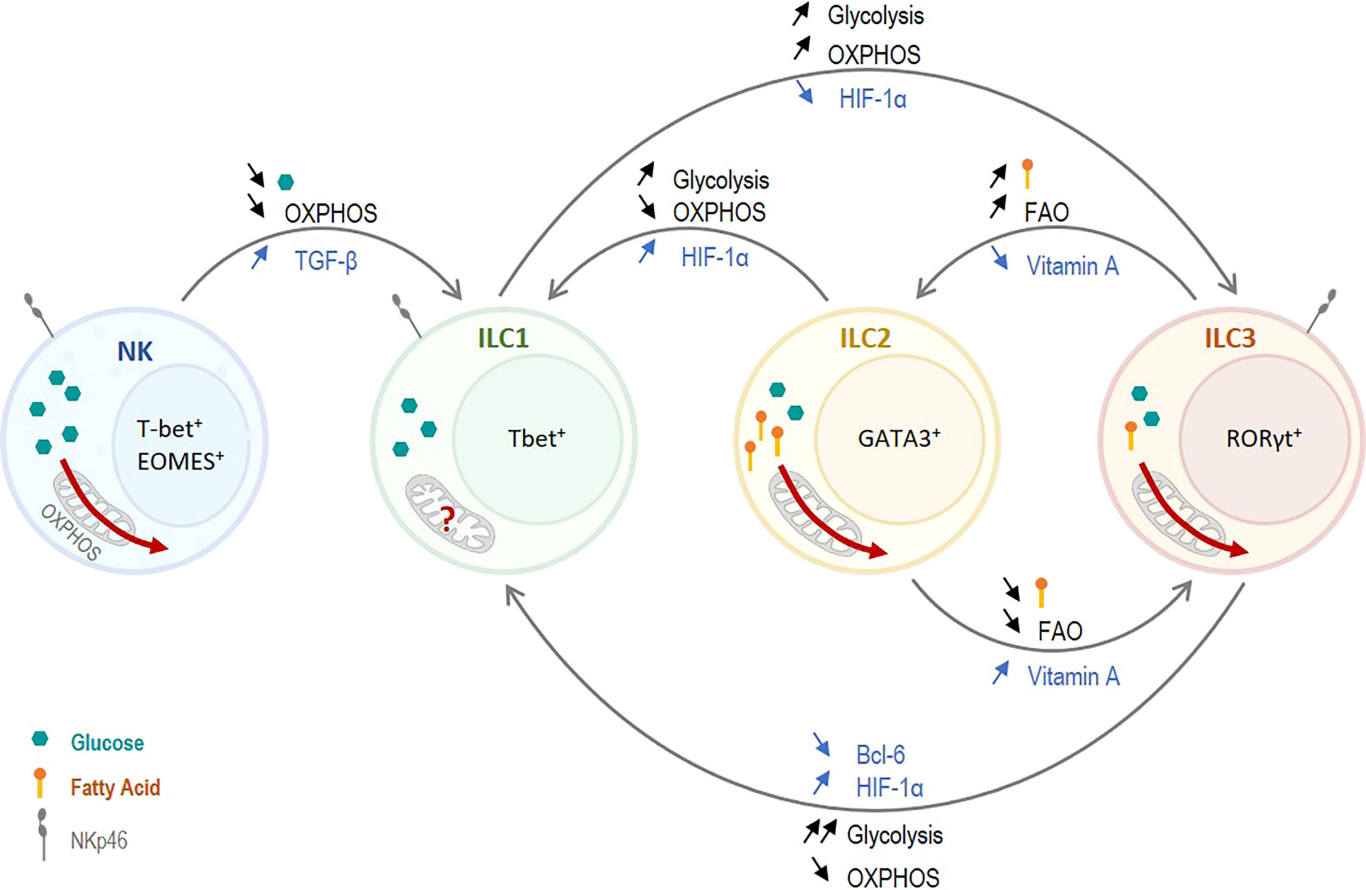
Role of Metabolism on ILC plasticity. Non-cytotoxic ILC1s have a glycolytic metabolism and presumably a low OXPHOS. Cytotoxic NK cells have high metabolic needs and rely on both OXPHOS and glycolysis. Thus, decreasing the overall metabolism, and especially OXPHOS, in NK cells promotes NK cell plasticity towards ILC1s. TGF-β was shown to induce this reduction in metabolism and to induce plasticity. Similarly, ILC2s and ILC3s strongly rely on OXPHOS, and a switch toward glycolysis promotes a conversion to ILC1s. On the other hand, increasing the use of OXPHOS in ILC1s induces a plasticity to ILC3-like cells. HIF-1α can trigger this conversion as it controls the balance between OXPHOS and glycolysis. ILC2s and ILC3s rely on glycolysis as well as OXPHOS. However, ILC2s use predominantly fatty acids, and fatty acid oxidation (FAO), to fuel OXPHOS. A tight control of the fatty acid uptake and the FAO play a critical role in the interconversion between ILC2s and ILC3s. Vitamin A, which reduces FAO, induces a plasticity of ILC2s toward ILC3s whereas vitamin A deficiency induces an ILC3-to-ILC2 plasticity.

These contrasting results may be due to the different deletion strategies that target HIF-1α in different ILC populations at different time points in ILC development. Fachi et al. targeted HIF-1α in NKp46^-^ and NKp46^+^ ILC3s but not ILC1s, while Krzywinska et al. targeted ILC1s and the NKp46^+^ ILC3 subset. It is therefore imaginable that HIF-1α has distinct roles in in NKp46^-^ and NKp46^+^ ILC3s. While HIF-1α plays a direct role in the transcription of RORγt and T-bet, it also plays a role in metabolism which may drive the interconversion of ILC1s and ILC3s. Indeed, ILC3s were shown to rely on glycolysis but also on fatty acid fuelled OXPHOS, and that glycolysis controls RORγt expression and cytokine production (58, 59). On the other hand, ILC1s are thought to rely almost only on glycolysis. Thus, a concomitant deletion of HIF-1a in both ILC1s and NKp46^+^ ILC3s might promote OXPHOS at the expense of glycolysis. Consequently, this could result in a switch away from glycolysis towards a more diverse metabolic profile in ILC1s during conversion into the NKp46^+^ ILC3 subset (**Figure 2**). Although, experimental evidence is still pending, it seems, hence, conceivable that NKp46^+^ ILC3s and NKp46^-^ ILC3s have a distinct metabolism. Moreover, in contrast to the study by Fachi et al., deletion of HIF-1α selectively in the NKp46^+^ ILC3 compartment becomes effective only after the acquisition of an ILC1-like phenotype, with IFN-γ production and reduced IL-22 expression (25, 30, 74). Therefore, the different deletion strategies target HIF1α at different time points of ILC development and metabolic needs are likely to change along this trajectory. Altogether, this may also explain why a deletion of the glycolytic driver HIF-1α in NKp46^+^ ILC3s versus NKp46^-^ ILC3s results in different outcomes.

As mentioned previously, the identity of ILCs depends mostly on their expression of lineage determining transcription factor T-bet, GATA3 and RORγt, and their secretion profile. Thus, plasticity between ILC1s and ILC3s is strongly dependent on the control of RORγt and T-bet expression. Indeed, IL-12 was shown to induce a conversion of gut ILC3s into ILC1s in humans by driving the downregulation of RORγt together with the upregulation of T-bet (16). The upregulation of T-bet leads to the production of IFN-γ by the ex-ILC3s, which complete their conversion into ILC1-like cells. Interestingly, it was shown that T-bet not only controls cytokine production but also controls glycolysis through different regulation pathways. Oestreich et al. have shown that T-bet, by repressing Bcl-6 transcription factor, upregulates glycolysis in murine Th1 cells (75). Indeed, Bcl-6 directly represses several genes of the glycolysis such as *Glut1* or *hexokinase 2* (75). Similarly, it was shown that loss of T-bet expression is correlated with a reduced mTORC1 activity and glycolysis in mice (76). mTORC1 is a master controller of glycolysis mainly by regulating transcription factor such as Srepb1 or HIF-1α which plays a key role in the regulation of OXPHOS and glycolysis (77). It was shown that a tight control of mTORC1-HIF-1α is important in the regulation of ILC3 metabolism and function (47, 58, 59). T-bet, by promoting glycolysis through the upregulation of mTORC1 and the inhibition of Bcl-6 promotes a metabolism strongly oriented toward glycolysis which resemble ILC1 metabolism. Thus, the upregulation of T-bet induced by cytokine stimulation *in vivo* and *in vitro* ILC3-to-ILC1 plasticity may be due partially to a strong upregulation of glycolysis in ILC3 at the expense of OXPHOS.

The differences in metabolism between ILC1s and ILC3s are subtle but play a key role in the distinction between the two subsets. While ILC1s seem to rely almost only on glycolysis, ILC3s need a balance between glycolysis and OXPHOS as decreasing one or the other impairs their development and effector function. Thus, a strong upregulation of glycolysis together with a strong downregulation of OXPHOS may promote ILC1-like phenotype. At the contrary, a mild upregulation of glycolysis which still allows OXPHOS, and the subsequent use of diverse carbon sources, may promote ILC3-like phenotype (**Figure 2**).

### ILC2 and ILC1

Plasticity of ILC2s toward ILC1-like cells was observed in *Mycobacterium tuberculosis (Mtb)*-infected mice in a pre-print study by Corral et al., which was driven by an upregulation of glycolysis in a HIF-1α-dependent manner (78). Type-1 inflammation induced by *Mtb* infection, which is recapitulated *in vitro* by IL-12 and IL-18 stimulation, leads to HIF-1α stabilization in all ILCs subsets in the lung. The stabilization of HIF-1α ILC2s has two effects, it upregulates genes associated with an ILC1 phenotype and switches the metabolism of ILC2s toward glycolysis (78). Upon *Mtb* infection, ILCs present in the lung become activated. As described above, the metabolic programs of ILC2s and ILC1s metabolism are quite distinct, ILC2s need to upregulate glycolysis as well as OXPHOS upon activation while ILC1s seem to rely mostly on glycolysis. However, the presence of HIF-1α in ILC2s induces a strong decrease in OXPHOS together with an increase glycolysis (Q. 55), which resembles ILC1 metabolism rather than baseline ILC2 metabolism. The induction of an ILC1-like metabolism in ILC2s may drive ILC2s towards an ILC1 phenotype as observed in *Mtb*-infected mice (**Figure 2**). This hypothesis is strongly supported by the fact that the conversion of ILC2s into ILC1-like cells and the production of IFN-γ was directly dependent on the upregulation of glycolysis (78). Although ILC1-to-ILC2 conversion remains to be formally established, it is perhaps tempting to speculate whether inhibiting glycolysis and boosting OXPHOS in ILC1s (e.g. by deleting HIF-1α) could facilitate an ILC2-like phenotype under distinct microenvironmental conditions.

*NK cells and ILC1s.* TGF-β has been shown to be the major regulator of NK cell to ILC1-like cell plasticity (20, 32). TGF-β is a regulatory molecule with multiple effects on several biological processes such as wound healing or immune response. TGF-β binds to the activin receptor like kinase 5 (ALK5) and TGFβ receptor II (TGFβRII) receptor complex to initiate its signalling cascade. ALK5 is activated and phosphorylates Smad proteins which regulates genes transcription (79, 80). TGF-β produced in the tumour microenvironment allows tumour to escape immunosurveillance by promoting the conversion of cytotoxic NK cells into non-cytotoxic ILC1-like cells (20). Interestingly, TGF-β has a significant impact on NK cells metabolism and effector function (81–84). The addition of TGF-β together with IL-2 has a strong impact on metabolic capacity and OXPHOS. It decreases the oxygen consumption in NK cells together with a strong decrease of the spare respiratory and the glycolytic capacity of these cells (83, 84). These results were confirmed by Slattery et al. who showed that anti-TGFβ increases oxygen consumption and spare respiratory capacity in NK cells from patient with breast cancer (81). Concomitantly, TGF-β also reduces IFN-γ production together with granzyme B and TRAIL which are key features of NK cell cytotoxicity (83, 84). However, the mechanisms by which TGF-β changes the metabolism of NK cells and the direct link with conversion of NK cells to ILC1s remain to be established. While mTORC1 seems to drive metabolic changes in NK cells from mice and patient with breast cancer, this is not the case in NK cells from healthy donors (81, 83, 84). Moreover, TGF-β has been shown to repress cMyc in different cell types, including T cells (85). As described above, cMyc is critical in the control of NK cell metabolism and function and its inhibition leads to a decreased glycolysis and OXPHOS (43).

Thus, TGF-β, by inhibiting mTOR and cMyc, strongly impairs NK cell metabolism which could lead to NK cell conversion from a cytotoxic effector cell to a non-cytotoxic ILC1-like cell. While the differences in metabolism between ILC1s and NK cells are not fully elucidated, it seems conceivable that NK cells have a higher metabolic need compared to ILC1s. Indeed, NK cells are cytotoxic and have a high energetic demand upon activation in order to produce and release cytokines as well as cytotoxic granules, and to directly kill using death ligands. That is probably why NK cells, when activated, maintain a high OXPHOS together with a strong upregulation of glycolysis. Thus, a drastic downregulation of OXPHOS and/or glycolysis may promote a non-cytotoxic ILC1-like phenotype which may require less energy (**Figure 2**).

### ILC2 and ILC3

ILCs of the gut, mostly ILC3s and ILC2s, are strongly influenced by food-derived metabolites and an excess or a deprivation in certain metabolites have an impact on gut homeostasis and ILCs balance. Vitamin A, as mentioned before, plays an important role in ILC3s homeostasis and was shown to influence ILC plasticity. Indeed, vitamin A-deficient mice were shown to have an increased number of ILC2s together with a decreased number of ILC3s (62). Interestingly, Goverse et al. showed that vitamin A deprivation affects both NKp46^+^ and NKp46^-^ ILC3 subsets (60). This switch in ILC balance toward ILC2s is accompanied by a reduced RORγt and type 3 cytokines production, together with an increased type 2 cytokine production (62). In contrast, supplementation of mice with vitamin A shows the reverse effect, an increased number of ILC3s and IL-22 together with a decreased number of ILC2s and IL-13 production (60, 62). In addition to directly inhibiting RORγt expression, vitamin A deprivation also strongly influences ILC metabolism. It was shown to significantly increase fatty acid uptakes and FAO in ILC2s and ILC3s but have no influence on glucose uptake and glycolysis (53). Once again, vitamin A supplementation was shown to have the reverse effect, a decrease in FAO and GATA3 expression in ILC2s (53, 62). At steady state, ILC2s and ILC3s from the gut have the same uptake of glucose which suppose the same glycolytic rate. However, ILC2s uptake significantly more fatty acids than ILC3s, which strongly indicates a higher FAO in ILC2s (53). Thus, vitamin A may control plasticity between ILC2 and ILC3 by a direct control of GATA3 and RORγt together with a switch of metabolism. Vitamin A decreases fatty acid uptake and FAO in ILC2s, promoting an ILC3-like metabolism. At the contrary, vitamin A deprivation increases fatty acids uptake and FAO in ILC3s which switches ILC3 metabolism toward an ILC2 metabolism (**Figure 2**). Surprisingly, Spencer at al. showed no plasticity between ILC2 and ILC3 when ILC2s were exposed to vitamin A or ILC3s to vitamin A inhibitor for three days (62). However, the complete media they used to culture the cells did not contain any fatty acids, preventing cells to use FAO. Thus, FAO being, in this case, the main trigger of vitamin A control of ILC balance, it may be interesting to repeat the same experiment including fatty acids so ILCs can use their full metabolic potential and not only be forced to use glucose as energy source.

## Conclusion

The last decade was important in the field of immunology as we discovered that metabolism is not only important in the energy supply of immune cells but also a key player in development, fate, effector function and plasticity of immune cells. ILCs are a relatively recently discovered immune cells type and we still strive to fully discover the metabolic programs that control ILC plasticity. Here, we aim to shed light on metabolic changes that may play an important role during the interconversion between ILC subsets. We describe distinct metabolic trajectories that occur during the interconversion of ILC subsets. While it is widely accepted that metabolic changes are required for ILC conversion, it remains to be determined whether a switch in metabolic program is sufficient to induce plasticity. Taken together, we suggest that even subtle changes in the balance between glycolytic rate and OXPHOS, along with discrete shifts in carbon sources, contribute to ILC plasticity.

## Author Contributions

AP and CS did literature research and wrote the manuscript. All authors contributed to the article and approved the submitted version.

## Conflict of Interest

The authors declare that the research was conducted in the absence of any commercial or financial relationships that could be construed as a potential conflict of interest.

## Publisher’s Note

All claims expressed in this article are solely those of the authors and do not necessarily represent those of their affiliated organizations, or those of the publisher, the editors and the reviewers. Any product that may be evaluated in this article, or claim that may be made by its manufacturer, is not guaranteed or endorsed by the publisher.
